# Supplementary material for: Impact of a High-Fat Diet on the Metabolomics Profile of 129S6 and C57BL6 Mouse Strains
Source: Int J Mol Sci. 2022 Oct 2;23(19):11682. doi: 10.3390/ijms231911682 (PMC9569783; doi:10.3390/ijms231911682)

Supplementary

Maria Piirsalu, Egon Taalberg, Mohan Jayaram, Kersti Lilleväli, Mihkel Zilmer and Eero Vasar

“Impact of a high-fat diet on the metabolomics profile of 129Sv and Bl6 mouse strains”

Supplementary Table S1

Two-way ANOVA [Diet (control diet or high-fat diet) x Strain (Bl6 or 129Sv)] summary table of high-fat diet (HFD)-induced changes in metabolite levels (log2 values. Mean ± SEM) and their ratios in Bl6 and 129Sv strain. Bonferroni post-hoc test was used for multiple comparisons. * Statistically significant difference between control and HFD in Bl6 or 129Sv groups; $^{+}$ Statistically significant difference between control diet groups. $^{@}$ Statistically significant difference between HFD groups. * p ≤ 0.05; ** p ≤ 0.01; *** p ≤ 0.001; **** p ≤ 0.0001

	**Bl6**		**129Sv**		**Two-way ANOVA**		
	**Control diet Mean ± SEM**	**HFD Mean ± SEM**	**Control diet Mean ± SEM**	**HFD Mean ± SEM**		***F***	***p***
**Acylcarnitines**							
Carnitine (C0)	4.50 ± 0.07	3.35 ± 0.09****	4.56 ± 0.10	3.02 ± 0.09****	**Diet**	226.1	<0.0001
					**Strain**	2.14	0.15
					**Diet X Strain**	4.59	0.04
Acetylcarnitine (C2)	3.48 ± 0.09	2.13 ± 0.112****	4.25 ± 0.18^{++}	2.17 ± 0.20****	**Diet**	118.9	<0.0001
					**Strain**	6.52	0.01
					**Diet X Strain**	5.51	0.02
Propionylcarnitine (C3)	-0.53 ± 0.06	-0.73 ± 0.05	-0.20 ± 0.05^{+++}	-0.57 ± 0.05****	**Diet**	28.76	<0.0001
					**Strain**	21.65	<0.0001
					**Diet X Strain**	2.31	0.14
Hydroxybutyryl-carnitine (C3-DC)	-1.83 ± 0.08	-1.82 ± 0.05	-1.60 ± 0.08	-1.75 ± 0.06	**Diet**	1.07	0.31
					**Strain**	4.54	0.04

					**Diet X Strain**	1.32	0.26
Hydroxypropionyl-carnitine (C3-OH)	-2.22 ± 0.04	-2.25 ± 0.06	-2.12 ± 0.05	-2.31 ± 0.09	**Diet**<br>**Strain**<br>**Diet X Strain**	3.07<br>0.13<br>1.81	0.09<br>0.72<br>0.19
Butyryl- and isobutyrylcarnitine (C4-)	-1.05 ± 0.09	-1.52 ± 0.05***	-0.59 ± 0.09^{+++}	-1.48 ± 0.04****	**Diet**<br>**Strain**<br>**Diet X Strain**	86.77<br>12.06<br>8.18	<0.0001<br>0.001<br>0.006
Propenoylcarnitine (C3:1)	-2.43 ± 0.06	-2.47 ± 0.07	-2.24 ± 0.06	-2.38 ± 0.08	**Diet**<br>**Strain**<br>**Diet X Strain**	1.74<br>4.11<br>0.49	0.19<br>0.05<br>0.49
Butenoylcarnitine (C4:1)	-1.76 ± 0.05	-1.89 ± 0.07	-1.58 ± 0.05	-1.92 ± 0.07**	**Diet**<br>**Strain**<br>**Diet X Strain**	13.95<br>1.41<br>2.81	0.0005<br>0.24<br>0.10
Isovalerylcarnitine and 2-methybutyrylcarnitine (C5-)	-1.33 ± 0.05	-1.33 ± 0.06	-1.12 ± 0.05	-1.25 ± 0.08	**Diet**<br>**Strain**<br>**Diet X Strain**	1.14<br>5.52<br>1.16	0.29<br>0.02<br>0.29
Methylglutarylcarnitine (C5-M-DC)	-3.18 ± 0.05	-3.21 ± 0.07	-3.08 ± 0.11	-3.14 ± 0.09	**Diet**<br>**Strain**<br>**Diet X Strain**	0.38<br>1.07<br>0.03	0.54<br>0.31<br>0.87
Glutarylcarnitine (C5-DC)	-3.13 ± 0.08	-3.19 ± 0.05	-2.98 ± 0.08	-3.15 ± 0.06	**Diet**<br>**Strain**<br>**Diet X Strain**	2.57<br>1.84<br>0.59	0.12<br>0.18<br>0.44
Hydroxyvaleryl-carnitine (C5-OH)	-2.89 ± 0.07	-2.98 ± 0.07	-2.94 ± 0.05	-3.02 ± 0.08	**Diet**<br>**Strain**<br>**Diet X Strain**	1.46<br>0.53<br>0.009	0.23<br>0.47<br>0.92
Tiglylcarnitine (C5:1)	-2.81 ± 0.05	-2.84 ± 0.05	-2.78 ± 0.07	-2.87 ± 0.07	**Diet**<br>**Strain**<br>**Diet X Strain**	0.86<br>0.0005<br>0.33	0.36<br>0.98<br>0.56
Glutaconylcarnitine (C5:1-DC)	-2.58 ± 0.07	-2.58 ± 0.05	-2.50 ± 0.07	-2.56 ± 0.07	**Diet**<br>**Strain**<br>**Diet X Strain**	0.25<br>0.67<br>0.22	0.62<br>0.42<br>0.64
Hexanoylcarnitine (C6)	-4.45 ± 0.04	-4.58 ± 0.06	-4.48 ± 0.07	-4.52 ± 0.04	**Diet**<br>**Strain**<br>**Diet X Strain**	2.37<br>0.05<br>0.78	0.13<br>0.83<br>0.38

Hexenoylcarnitine (C6:1)	-5.14 ± 0.06	-5.12 ± 0.04	-5.12 ± 0.06	-5.14 ± 0.04	**Diet**<br>**Strain**<br>**Diet X Strain**	0.01<br>0.003<br>0.15	0.92<br>0.96<br>0.70
Pimelylcarnitine (C7-DC)	-3.86 ± 0.06	-3.72 ± 0.08	-3.65 ± 0.08	-3.63 ± 0.04	**Diet**<br>**Strain**<br>**Diet X Strain**	1.36<br>5.15<br>0.86	0.25<br>0.03<br>0.36
Octanoylcarnitine (C8)	-2.55 ± 0.06	-2.61 ± 0.05	-2.53 ± 0.06	-2.45 ± 0.05	**Diet**<br>**Strain**<br>**Diet X Strain**	0.04<br>2.94<br>1.64	0.85<br>0.09<br>0.21
Nonanoylcarnitine (C9)	-3.27 ± 0.12	-3.09 ± 0.08	-3.07 ± 0.03	-3.14 ± 0.07	**Diet**<br>**Strain**<br>**Diet X Strain**	0.51<br>0.86<br>2.56	0.48<br>0.36<br>0.12
Decanoylcarnitine (C10)	-2.35 ± 0.10	-2.34 ± 0.07	-2.34 ± 0.06	-2.42 ± 0.09	**Diet**<br>**Strain**<br>**Diet X Strain**	0.15<br>0.17<br>0.36	0.70<br>0.68<br>0.55
Decenoylcarnitine (C10:1)	-3.65 ± 0.07	-3.58 ± 0.07	-3.50 ± 0.06	-3.58 ± 0.04	**Diet**<br>**Strain**<br>**Diet X Strain**	0.002<br>1.53<br>1.69	0.96<br>0.22<br>0.20
Decadienyl-carnitine (C10:2)	-3.40 ± 0.05	-3.31 ± 0.07	-3.25 ± 0.06	3.45 ± 0.08	**Diet**<br>**Strain**<br>**Diet X Strain**	0.66<br>0.002<br>5.09	0.42<br>0.96<br>0.03
Dodecanoyl-carnitine (C12)	-3.64 ± 0.05	-3.67 ± 0.08	-3.24 ± 0.12++	-3.65 ± 0.05**	**Diet**<br>**Strain**<br>**Diet X Strain**	7.20<br>6.61<br>5.62	0.01<br>0.01<br>0.02
Dodecanedioyl-carnitine C12-DC	-1.88 ± 0.07	-1.88 ± 0.07	-1.89 ± 0.04	-2.00 ± 0.05	**Diet**<br>**Strain**<br>**Diet X Strain**	0.87<br>1.08<br>0.81	0.36<br>0.30<br>0.37
Dodecenoyl-carnitine (C12:1)	-3.50 ± 0.08	-3.69 ± 0.08	-3.14 ± 0.11+	-3.53 ± 0.08*	**Diet**<br>**Strain**<br>**Diet X Strain**	10.94<br>8.93<br>1.33	0.002<br>0.005<br>0.25
Tetradecanoyl-carnitine (C14)	-3.54 ± 0.08	-3.89 ± 0.08	-3.01 ± 0.22+	-3.65 ± 0.11*	**Diet**<br>**Strain**<br>**Diet X Strain**	12.94<br>8.04<br>1.13	0.0008<br>0.007<br>0.29
					**Diet**	5.18	0.03

Tetradecenoyl-carnitine (C14:1)	-3.96 ± 0.17	-4.23 ± 0.14	-3.53 ± 0.19	-3.98 ± 0.10	**Strain**<br>**Diet X Strain**	4.53<br>0.29	0.04<br>0.59
Hydroxytetra-decenoylcarnitine (C14:1-OH)	-5.07 ± 0.10	-4.98 ± 0.09	-4.65 ± 0.14	-4.96 ± 0.11	**Diet**<br>**Strain**<br>**Diet X Strain**	0.97<br>3.64<br>3.26	0.33<br>0.06<br>0.08
Tetradecadienyl-carnitine (C14:2)	-4.85 ± 0.10	-5.06 ± 0.15	-4.37 ± 0.13^{+}	-5.17 ± 0.05****	**Diet**<br>**Strain**<br>**Diet X Strain**	19.03<br>2.54<br>6.74	<0.0001<br>0.12<br>0.01
Hydroxytetra-decadienylcarnitine C14:2-OH	-6.86 ± 0.07	-6.95 ± 0.09	-6.70 ± 0.09	-6.80 ± 0.11	**Diet**<br>**Strain**<br>**Diet X Strain**	1.01<br>2.91<br>0.001	0.32<br>0.10<br>0.97
Hexadecanoyl-carnitine (C16)	-2.71 ± 0.11	-2.92 ± 0.10	-1.84 ± 0.26^{++}	-2.45 ± 0.11	**Diet**<br>**Strain**<br>**Diet X Strain**	6.09<br>16.48<br>1.46	0.02<br>0.0002<br>0.23
Hydroxyhexa-decanoylcarnitine (C16-OH)	-4.50 ± 0.13	-4.54 ± 0.06	-4.27 ± 0.10	-4.50 ± 0.10	**Diet**<br>**Strain**<br>**Diet X Strain**	1.81<br>1.88<br>0.84	0.19<br>0.18<br>0.37
Hexadecenoyl-carnitine (C16:1)	-3.94 ± 0.09	-4.37 ± 0.11	-3.43 ± 0.22	-4.27 ± 0.11**	**Diet**<br>**Strain**<br>**Diet X Strain**	18.11<br>4.12<br>1.81	0.0001<br>0.05<br>0.19
Hydroxyhexa-decenoylcarnitine (C16:1-OH)	-6.75 ± 0.10	-6.70 ± 0.22	-6.53 ± 0.11	-6.70 ± 0.12	**Diet**<br>**Strain**<br>**Diet X Strain**	0.19<br>0.72<br>0.66	0.66<br>0.40<br>0.42
Hexadecadienyl-carnitine C16:2	-4.31 ± 0.05	-4.45 ± 0.04	-4.30 ± 0.08	-4.55 ± 0.05*	**Diet**<br>**Strain**<br>**Diet X Strain**	10.92<br>0.60<br>0.79	0.002<br>0.44<br>0.38
Hydroxyhexa-decadienylcarnitine (C16:2-OH)	-4.24 ± 0.10	-4.30 ± 0.06	-4.11 ± 0.08	-4.57 ± 0.09**	**Diet**<br>**Strain**<br>**Diet X Strain**	9.73<br>0.67<br>5.63	0.003<br>0.42<br>0.02
Octadecanoylcarnitine (C18)	-4.10 ± 0.08	-3.57 ± 0.05**	-3.72 ± 0.14	-3.09 ± 0.12***$^{@}$	**Diet**<br>**Strain**<br>**Diet X Strain**	31.10<br>16.85<br>0.28	<0.0001<br>0.0002<br>0.60
Octadecenoyl-carnitine (C18:1)	-3.58 ± 0.13	-3.57 ± 0.11	-3.22 ± 0.19	-3.10 ± 0.17	**Diet**<br>**Strain**	0.19<br>7.55	0.66<br>0.009

					**Diet X Strain**	0.15	0.71
Hydroxyocta-decenoylcarnitine (C18:1-OH)	-4.46 ± 0.08	-4.56 ± 0.08	-4.10 ± 0.06^{+}	-4.53 ± 0.12**	**Diet**<br>**Strain**<br>**Diet X Strain**	8.63<br>4.79<br>3.48	0.005<br>0.03<br>0.07
Octadecadienyl-carnitine (C18:2)	-5.18 ± 0.11	-5.95 ± 0.10**	-4.52 ± 0.21^{+}	-5.70 ± 0.12****	**Diet**<br>**Strain**<br>**Diet X Strain**	41.68<br>9.23<br>1.82	<0.0001<br>0.004<br>0.18
**Amino Acids**							
Alanine (Ala)	8.15 ± 0.05	8.27 ± 0.11	8.21 ± 0.08	7.97 ± 0.15	**Diet**<br>**Strain**<br>**Diet X Strain**	0.38<br>1.31<br>2.98	0.54<br>0.26<br>0.09
Arginine (Arg)	6.20 ± 0.14	6.16 ± 0.16	6.82 ± 0.15^{+}	6.28 ± 0.15	**Diet**<br>**Strain**<br>**Diet X Strain**	3.91<br>6.14<br>2.81	0.05<br>0.02<br>0.10
Asparagine (Asn)	5.12 ± 0.14	5.20 ± 0.14	5.36 ± 0.09	5.14 ± 0.17	**Diet**<br>**Strain**<br>**Diet X Strain**	0.26<br>0.41<br>1.16	0.61<br>0.53<br>0.29
Aspartate (Asp)	5.66 ± 0.12	5.24 ± 0.22	5.99 ± 0.19	5.69 ± 0.14	**Diet**<br>**Strain**<br>**Diet X Strain**	3.78<br>4.50<br>0.12	0.06<br>0.04<br>0.73
Citrulline (Cit)	5.41 ± 0.06	5.74 ± 0.09*	5.73 ± 0.09^{+}	5.67 ± 0.09	**Diet**<br>**Strain**<br>**Diet X Strain**	2.62<br>2.40<br>5.78	0.11<br>0.13<br>0.02
Glutamine (Gln)	9.37 ± 0.06	9.12 ± 0.07	9.04 ± 0.08^{+}	9.12 ± 0.12	**Diet**<br>**Strain**<br>**Diet X Strain**	1.00<br>3.64<br>3.86	0.32<br>0.06<br>0.06
Glutamate (Glu)	7.26 ± 0.26	7.09 ± 0.21	7.37 ± 0.15	7.50 ± 0.16	**Diet**<br>**Strain**<br>**Diet X Strain**	0.01<br>1.73<br>0.59	0.93<br>0.19<br>0.45
Glycine (Gly)	7.68 ± 0.07	7.41 ± 0.10	7.95 ± 0.09	7.20 ± 0.05****	**Diet**<br>**Strain**<br>**Diet X Strain**	40.05<br>0.19<br>8.84	<0.0001<br>0.67<br>0.005

Histidine (His)	5.74 ± 0.05	6.04 ± 0.09	5.96 ± 0.04	6.09 ± 0.10	**Diet**	8.33	0.006
					**Strain**	3.34	0.07
					**Diet X Strain**	1.08	0.30
Isoleucine (Ile)	6.22 ± 0.10	6.62 ± 0.19	6.99 ± 0.08^{++}	7.48 ± 0.15$^{@@@}$	**Diet**	10.70	0.002
					**Strain**	35.75	<0.0001
					**Diet X Strain**	0.12	0.73
Leucine (Leu)	7.04 ± 0.08	7.38 ± 0.15	7.68 ± 0.08^{++}	8.08 ± 0.14$^{@@@}$	**Diet**	10.34	0.002
					**Strain**	34.63	<0.0001
					**Diet X Strain**	0.08	0.77
Lysine (Lys)	7.48 ± 0.11	7.99 ± 0.16	8.38 ± 0.14^{+++}	8.10 ± 0.15	**Diet**	0.71	0.40
					**Strain**	13.35	0.0007
					**Diet X Strain**	8.35	0.006
Methionine (Met)	5.59 ± 0.12	5.96 ± 0.20	6.31 ± 0.20^{+}	6.01 ± 0.21	**Diet**	0.03	0.85
					**Strain**	4.35	0.04
					**Diet X Strain**	3.21	0.08
Ornithine (Orn)	5.45 ± 0.12	4.93 ± 0.17	6.14 ± 0.10^{++}	5.70 ± 0.13$^{@@}$	**Diet**	13.22	0.0007
					**Strain**	30.28	<0.0001
					**Diet X Strain**	0.09	0.76
Phenylalanine (Phe)	5.96 ± 0.07	5.82 ± 0.09	6.30 ± 0.08	6.25 ± 0.11$^{@@}$	**Diet**	1.15	0.29
					**Strain**	18.85	<0.0001
					**Diet X Strain**	0.26	0.61
Proline (Pro)	6.41 ± 0.12	6.69 ± 0.23	6.60 ± 0.13	6.61 ± 0.22	**Diet**	0.67	0.42
					**Strain**	0.09	0.76
					**Diet X Strain**	0.56	0.46
Serine (Ser)	6.52 ± 0.10	6.58 ± 0.14	7.11 ± 0.08^{++}	6.81 ± 0.13	**Diet**	1.20	0.28
					**Strain**	13.53	0.0006
					**Diet X Strain**	2.45	0.12
Tryptophan (Trp)	6.17 ± 0.08	5.87 ± 0.08	6.42 ±0.06	6.17 ± 0.10	**Diet**	12.32	0.001
					**Strain**	12.54	0.0009
					**Diet X Strain**	0.11	0.75
Tyrosine (Tyr)	5.84 ± 0.13	5.93 ± 0.14	5.93 ± 0.11	6.26 ± 0.18	**Diet**	2.19	0.15
					**Strain**	2.19	0.15
					**Diet X Strain**	0.74	0.39
Valine (Val)					**Diet**	18.55	<0.0001

	7.41 ± 0.07	7.95 ± 0.21	8.05 ± 0.09^{+}	8.71 ± 0.16**$^{@@}$	**Strain**<br>**Diet X Strain**	25.59<br>0.21	<0.0001<br>0.65
**Biogenic Amines**							
Asymmetric dimethylarginine (ADMA)	-1.32 ± 0.15	-1.45 ± 0.14	-1.15 ± 0.17	-1.15 ± 0.12	**Diet**<br>**Strain**<br>**Diet X Strain**	0.19<br>2.26<br>0.16	0.66<br>0.14<br>0.69
Alpha-Aminoadipic acid (Alpha-AAA)	1.73 ± 0.11	2.02 ± 0.11	1.11 ± 0.07^{++}	0.91 ± 0.05$^{@@@@}$	**Diet**<br>**Strain**<br>**Diet X Strain**	0.15<br>57.02<br>4.78	0.70<br><0.0001<br>0.04
Carnosine	2.26 ± 0.08	2.17 ± 0.18	0.77 ± 0.15^{++++}	0.79 ± 0.14$^{@@@@}$	**Diet**<br>**Strain**<br>**Diet X Strain**	0.07<br>96.09<br>0.16	0.79<br><0.0001<br>0.69
Creatinine	2.93 ± 0.06	2.83 ± 0.06	3.06 ± 0.08	2.91 ± 0.08	**Diet**<br>**Strain**<br>**Diet X Strain**	3.22<br>2.15<br>0.09	0.08<br>0.15<br>0.77
Histamine	-0.78 ± 0.14	-0.76 ± 0.08	-0.60 ± 0.07	-0.82 ± 0.09	**Diet**<br>**Strain**<br>**Diet X Strain**	1.11<br>0.31<br>1.46	0.30<br>0.58<br>0.23
Putrescine	-0.42 ± 0.12	-0.12 ± 0.14	-0.83 ± 0.16	-1.12 ± 0.11$^{@@@@}$	**Diet**<br>**Strain**<br>**Diet X Strain**	0.005<br>26.63<br>4.66	0.95<br><0.0001<br>0.04
Serotonin (5-HT)	3.05 ± 0.37	3.47 ± 0.36	3.11 ± 0.20	3.18 ± 0.19	**Diet**<br>**Strain**<br>**Diet X Strain**	0.78<br>0.16<br>0.39	0.39<br>0.69<br>0.54
Spermidine	0.93 ± 0.10	0.93 ± 0.09	1.26 ± 0.14	0.83 ± 0.08*	**Diet**<br>**Strain**<br>**Diet X Strain**	4.05<br>1.32<br>4.21	0.05<br>0.26<br>0.05
Spermine	-0.47 ± 0.13	-0.55 ± 0.12	-0.40 ± 0.12	-0.50 ± 0.17	**Diet**<br>**Strain**<br>**Diet X Strain**	0.44<br>0.19<br>0.01	0.51<br>0.67<br>0.92
					**Diet**	5.79	0.02

Trans-4-Hydroxyproline (t4-OH-Pro)	3.23 ± 0.08	3.46 ± 0.07	3.27 ± 0.08	2.69 ± 0.06****@@@@	**Strain**<br>**Diet X Strain**	24.55<br>31.77	<0.0001<br><0.0001
Kynurenine	0.27 ± 0.11	-0.61 ± 0.15****	0.68 ± 0.08	-0.05 ± 0.13***@	**Diet**<br>**Strain**<br>**Diet X Strain**	46.38<br>16.81<br>0.42	<0.0001<br>0.0002<br>0.52
Methionine sulfoxide (Met-SO)	-0.04 ± 0.52	1.19 ± 0.68	1.30 ± 0.28	1.80 ± 0.33	**Diet**<br>**Strain**<br>**Diet X Strain**	3.60<br>4.59<br>0.62	0.06<br>0.04<br>0.44
Nitro-Tyrosine	-0.35 ± 0.08	-0.40 ± 0.13	-0.09 ± 0.04	-0.14 ± 0.07	**Diet**<br>**Strain**<br>**Diet X Strain**	0.38<br>8.88<br>0.00	0.54<br>0.006<br>0.98
cis-4-Hydroxyproline (c4-OH-Pro)	-1.61 ± 0.49	-1.85 ± 0.32	-2.29 ± 0.11	-2.97 ± 0.00	**Diet**<br>**Strain**<br>**Diet X Strain**	1.70<br>6.50<br>0.40	0.22<br>0.03<br>0.54
**Glycerophospholipids**							
Lysophosphatidylcholine acyls							
lysoPC a C14:0	2.81 ± 0.09	2.59 ± 0.14	2.73 ± 0.12	2.81 ± 0.162	**Diet**<br>**Strain**<br>**Diet X Strain**	0.27<br>0.27<br>1.15	0.61<br>0.61<br>0.29
lysoPC a C16:0	7.08 ± 0.07	7.27 ± 0.07	7.13 ± 0.06	7.03 ± 0.08	**Diet**<br>**Strain**<br>**Diet X Strain**	0.40<br>1.72<br>4.43	0.53<br>0.20<br>0.04
lysoPC a C16:1	2.45 ± 0.11	2.46 ± 0.14	1.51 ± 0.10++++	1.44 ± 0.13@@@@	**Diet**<br>**Strain**<br>**Diet X Strain**	0.07<br>65.22<br>0.09	0.79<br><0.0001<br>0.77
lysoPC a C17:0	0.90 ± 0.07	0.80 ± 0.06	1.53 ± 0.06++++	1.12 ± 0.09***@	**Diet**<br>**Strain**<br>**Diet X Strain**	13.54<br>46.48<br>5.06	0.0006<br><0.0001<br>0.03
lysoPC a C18:0	5.91 ± 0.08	6.56 ± 0.08****	6.05 ± 0.05	6.61 ± 0.07****	**Diet**<br>**Strain**<br>**Diet X Strain**	72.70<br>1.57<br>0.46	<0.0001<br>0.22<br>0.50

lysoPC a C18:1	5.02 ± 0.10	6.36 ± 0.12****	4.55 ± 0.08^{+}	6.11 ± 0.14****	**Diet**<br>**Strain**<br>**Diet X Strain**	168.24<br>10.50<br>1.04	<0.0001<br>0.002<br>0.31
lysoPC a C18:2	6.31 ± 0.08	6.79 ± 0.12*	6.24 ± 0.06	6.81 ± 0.14**	**Diet**<br>**Strain**<br>**Diet X Strain**	27.07<br>0.05<br>0.21	<0.0001<br>0.82<br>0.65
lysoPC a C20:3	2.40 ± 0.16	4.13 ± 0.09****	1.56 ± 0.09^{++++}	3.50 ± 0.11****$^{@@}$	**Diet**<br>**Strain**<br>**Diet X Strain**	255.48<br>41.19<br>0.91	<0.0001<br><0.0001<br>0.35
lysoPC a C20:4	4.46 ± 0.10	4.67 ± 0.10	4.10 ± 0.06	4.59 ± 0.14**	**Diet**<br>**Strain**<br>**Diet X Strain**	12.08<br>4.61<br>1.85	0.001<br>0.04<br>0.18
lysoPC a C24:0	-0.09 ± 0.09	0.05 ± 0.07	0.44 ± 0.05^{++++}	0.47 ± 0.10$^{@@}$	**Diet**<br>**Strain**<br>**Diet X Strain**	1.19<br>38.20<br>0.47	0.28<br><0.0001<br>0.49
lysoPC a C26:0	-2.54 ± 0.12	-1.86 ± 0.08****	-2.50 ± 0.07	-1.95 ± 0.11***	**Diet**<br>**Strain**<br>**Diet X Strain**	42.23<br>0.09<br>0.47	<0.0001<br>0.77<br>0.50
lysoPC a C26:1	-1.78 ± 0.14	-1.38 ± 0.12	-1.50 ± 0.11	-1.26 ± 0.14	**Diet**<br>**Strain**<br>**Diet X Strain**	6.18<br>2.57<br>0.43	0.02<br>0.11<br>0.51
lysoPC a C28:0	-1.74 ± 0.27	-1.91 ± 0.09	-2.02 ± 0.22	-1.77 ± 0.13	**Diet**<br>**Strain**<br>**Diet X Strain**	0.05<br>0.14<br>1.23	0.82<br>0.71<br>0.27
lysoPC a C28:1	-2.24 ± 0.10	-1.67 ± 0.09***	-2.33 ± 0.05	-1.60 ± 0.11****	**Diet**<br>**Strain**<br>**Diet X Strain**	54.37<br>0.006<br>0.81	<0.0001<br>0.94<br>0.37
Phosphatidylcholine diacyls							
PC aa C24:0	-2.63 ± 0.09	-2.39 ± 0.14	-2.60 ± 0.12	-2.40 ± 0.13	**Diet**<br>**Strain**<br>**Diet X Strain**	3.30<br>0.01<br>0.03	0.08<br>0.92<br>0.86
PC aa C26:0	-1.07 ± 0.10	-0.86 ± 0.07	-1.09 ± 0.06	-0.76 ± 0.09*	**Diet**<br>**Strain**	11.09<br>0.23	0.002<br>0.63

					Diet X Strain	0.57	0.45
PC aa C28:1	-1.85 ± 0.08	-1.13 ± 0.07****	-1.71 ± 0.06	-0.78 ± 0.07****$^{@@}$	**Diet**	152.02	<0.0001
					**Strain**	13.89	0.0005
					**Diet X Strain**	2.44	0.13
PC aa C30:0	0.24 ± 0.14	0.98 ± 0.10**	0.65 ± 0.16	0.89 ± 0.13	**Diet**	12.82	0.0008
					**Strain**	1.39	0.24
					**Diet X Strain**	3.45	0.07
PC aa C30:2	-6.43 ± 0.07	-5.53 ± 0.07****	-5.98 ± 0.05^{++++}	-5.11 ± 0.06****$^{@@@@}$	**Diet**	211.25	<0.0001
					**Strain**	51.16	<0.0001
					**Diet X Strain**	0.04	0.84
PC aa C32:0	3.86 ± 0.10	4.14 ± 0.07	4.35 ± 0.13^{+}	4.05 ± 0.12	**Diet**	0.0009	0.98
					**Strain**	3.25	0.08
					**Diet X Strain**	6.86	0.01
PC aa C32:1	3.35 ± 0.12	4.13 ± 0.07***	3.35 ± 0.16	3.38 ± 0.14$^{@@@}$	**Diet**	9.75	0.003
					**Strain**	8.21	0.006
					**Diet X Strain**	8.21	0.006
PC aa C32:2	-0.55 ± 0.07	-0.35 ± 0.07	-0.51 ± 0.08	-0.43 ± 0.06	**Diet**	4.01	0.05
					**Strain**	0.07	0.79
					**Diet X Strain**	0.80	0.38
PC aa C32:3	-4.06 ± 0.07	-3.67 ± 0.06**	-3.78 ± 0.06^{+}	-3.30 ± 0.07****$^{@@@}$	**Diet**	40.65	<0.0001
					**Strain**	23.10	<0.0001
					**Diet X Strain**	0.46	0.50
PC aa C34:1	6.88 ± 0.10	8.75 ± 0.08****	7.01 ± 0.10	8.52 ± 0.14****	**Diet**	251.74	<0.0001
					**Strain**	0.20	0.65
					**Diet X Strain**	2.71	0.11
PC aa C34:2	8.19 ± 0.08	8.68 ± 0.07***	8.59 ± 0.07^{++}	8.70 ± 0.08	**Diet**	16.40	0.0002
					**Strain**	8.03	0.007
					**Diet X Strain**	6.62	0.01
PC aa C34:3	4.05 ± 0.08	4.02 ± 0.08	3.63 ± 0.09^{++}	3.37 ± 0.09$^{@@@@}$	**Diet**	2.85	0.10
					**Strain**	38.23	<0.0001
					**Diet X Strain**	1.81	0.19
PC aa C34:4	-0.57 ± 0.08	-0.38 ± 0.09	-0.89 ± 0.07^{+}	-0.91 ± 0.10$^{@@@}$	**Diet**	1.10	0.30
					**Strain**	26.69	<0.0001
					**Diet X Strain**	1.62	0.21

PC aa C36:0	0.63 ± 0.05	0.39 ± 0.15	1.12 ± 0.08++	0.80 ± 0.10@	**Diet**<br>**Strain**<br>**Diet X Strain**	9.61<br>12.60<br>0.72	0.003<br>0.0009<br>0.40
PC aa C36:1	4.29 ± 0.12	7.31 ± 0.10****	4.38 ± 0.09	7.36 ± 0.08****	**Diet**<br>**Strain**<br>**Diet X Strain**	618.63<br>1.47<br>0.23	<0.0001<br>0.23<br>0.64
PC aa C36:2	7.38 ± 0.07	8.81 ± 0.07****	7.93 ± 0.05++++	9.12 ± 0.06****@@	**Diet**<br>**Strain**<br>**Diet X Strain**	440.90<br>47.39<br>3.40	<0.0001<br><0.0001<br>0.07
PC aa C36:3	6.37 ± 0.08	7.71 ± 0.08****	6.30 ± 0.05	7.43 ± 0.08****	**Diet**<br>**Strain**<br>**Diet X Strain**	304.61<br>5.89<br>2.15	<0.0001<br>0.02<br>0.15
PC aa C36:4	7.18 ± 0.08	7.63 ± 0.08***	7.41 ± 0.05	7.60 ± 0.08	**Diet**<br>**Strain**<br>**Diet X Strain**	19.88<br>2.01<br>3.43	<0.0001<br>0.16<br>0.07
PC aa C36:5	2.98 ± 0.09	3.17 ± 0.08	2.70 ± 0.06	2.65 ± 0.10@@@	**Diet**<br>**Strain**<br>**Diet X Strain**	0.79<br>23.19<br>2.05	0.38<br><0.0001<br>0.16
PC aa C36:6	-1.31 ± 0.09	-1.25 ± 0.08	-1.39 ± 0.08	-1.47 ± 0.08	**Diet**<br>**Strain**<br>**Diet X Strain**	0.01<br>3.18<br>0.79	0.91<br>0.08<br>0.38
PC aa C38:0	0.38 ± 0.07	0.80 ± 0.05****	0.72 ± 0.06++	1.05 ± 0.06**@	**Diet**<br>**Strain**<br>**Diet X Strain**	38.76<br>23.86<br>0.50	<0.0001<br><0.0001<br>0.48
PC aa C38:1	-2.70 ± 0.98	0.13 ± 0.27**	-2.10 ± 0.47	0.45 ± 0.16**	**Diet**<br>**Strain**<br>**Diet X Strain**	29.48<br>0.87<br>0.07	<0.0001<br>0.36<br>0.79
PC aa C38:3	4.55 ± 0.10	6.07 ± 0.07****	4.79 ± 0.04	5.90 ± 0.07****	**Diet**<br>**Strain**<br>**Diet X Strain**	311.10<br>0.24<br>7.77	<0.0001<br>0.63<br>0.008
PC aa C38:4	6.28 ± 0.08	6.95 ± 0.11****	6.62 ± 0.07	7.13 ± 0.10**	**Diet**<br>**Strain**<br>**Diet X Strain**	41.13<br>7.98<br>0.75	<0.0001<br>0.007<br>0.39
PC aa C38:5					**Diet**	60.13	<0.0001

	5.05 ± 0.11	5.67 ± 0.09****	5.15 ± 0.06	5.81 ± 0.07****	**Strain**<br>**Diet X Strain**	2.15<br>0.05	0.15<br>0.82
PC aa C38:6	6.63 ± 0.09	6.50 ± 0.08	6.95 ± 0.05^{+}	6.70 ± 0.09	**Diet**<br>**Strain**<br>**Diet X Strain**	6.15<br>10.61<br>0.56	0.02<br>0.002<br>0.46
PC aa C40:1	-2.00 ± 0.09	-1.93 ± 0.07	-1.79 ± 0.09	-1.72 ± 0.05	**Diet**<br>**Strain**<br>**Diet X Strain**	0.82<br>7.50<br>0.01	0.37<br>0.009<br>0.94
PC aa C40:2	-1.91 ± 0.12	-1.68 ± 0.08	-1.28 ± 0.05^{++++}	-1.31 ± 0.09$^{@}$	**Diet**<br>**Strain**<br>**Diet X Strain**	1.27<br>32.18<br>2.05	0.26<br><0.0001<br>0.16
PC aa C40:3	-0.84 ± 0.09	-0.22 ± 0.09****	-0.68 ± 0.07	-0.27 ± 0.05**	**Diet**<br>**Strain**<br>**Diet X Strain**	43.94<br>0.49<br>2.00	<0.0001<br>0.49<br>0.16
PC aa C40:4	1.64 ± 0.10	2.17 ± 0.09****	2.02 ± 0.06^{+}	2.34 ± 0.07*	**Diet**<br>**Strain**<br>**Diet X Strain**	30.19<br>12.43<br>2.08	<0.0001<br>0.001<br>0.16
PC aa C40:5	2.23 ± 0.12	2.92 ± 0.11****	2.81 ± 0.06^{+++}	3.53 ± 0.08****$^{@@@}$	**Diet**<br>**Strain**<br>**Diet X Strain**	56.61<br>40.04<br>0.02	<0.0001<br><0.0001<br>0.88
PC aa C40:6	4.85 ± 0.09	4.97 ± 0.08	5.42 ± 0.07^{+++}	5.34 ± 0.10$^{@}$	**Diet**<br>**Strain**<br>**Diet X Strain**	0.06<br>30.60<br>1.43	0.81<br><0.0001<br>0.24
PC aa C42:0	-2.67 ± 0.09	-2.55 ± 0.12	-2.22 ± 0.10^{+}	-2.40 ± 0.09	**Diet**<br>**Strain**<br>**Diet X Strain**	0.07<br>8.53<br>1.99	0.79<br>0.006<br>0.17
PC aa C42:1	-2.83 ± 0.10	-2.83 ± 0.09	-2.53 ± 0.08	-2.62 ± 0.13	**Diet**<br>**Strain**<br>**Diet X Strain**	0.22<br>6.44<br>0.19	0.64<br>0.01<br>0.67
PC aa C42:2	-2.27 ± 0.10	-2.17 ± 0.05	-1.73 ± 0.09^{++++}	-2.14 ± 0.06**	**Diet**<br>**Strain**<br>**Diet X Strain**	3.75<br>12.57<br>10.07	0.06<br>0.0009<br>0.003
PC aa C42:4	-2.47 ± 0.06	-2.53 ± 0.11	-2.15 ± 0.08^{+}	-2.28 ± 0.08	**Diet**<br>**Strain**	1.25<br>11.97	0.27<br>0.001

					**Diet X Strain**	0.17	0.69
PC aa C42:5	-1.76 ± 0.11	-1.92 ± 0.06	-1.25 ± 0.08+++	-1.69 ± 0.10**	**Diet**<br>**Strain**<br>**Diet X Strain**	10.81<br>17.12<br>2.61	0.002<br>0.0002<br>0.11
PC aa C42:6	-0.04 ± 0.08	-0.27 ± 0.09	0.36 ± 0.08++	0.12 ± 0.07@	**Diet**<br>**Strain**<br>**Diet X Strain**	8.25<br>22.98<br>0.01	0.006<br><0.0001<br>0.93
Phosphatidylcholine acyl-alkyls							
PC ae C30:0	-2.56 ± 0.07	-2.33 ± 0.03*	-2.38 ± 0.04	-2.26 ± 0.05	**Diet**<br>**Strain**<br>**Diet X Strain**	12.43<br>6.67<br>1.19	0.001<br>0.01<br>0.28
PC ae C30:1	-4.07 ± 0.12	-3.89 ± 0.19	-4.06 ± 0.24	-3.73 ± 0.29	**Diet**<br>**Strain**<br>**Diet X Strain**	1.33<br>0.15<br>0.11	0.25<br>0.70<br>0.74
PC ae C30:2	-1.95 ± 0.05	-1.35 ± 0.08	-1.38 ± 0.06	-2.79 ± 0.33****@@@@	**Diet**<br>**Strain**<br>**Diet X Strain**	5.62<br>6.45<br>34.76	0.02<br>0.01<br><0.0001
PC ae C32:1	-0.36 ± 0.06	0.12 ± 0.07****	0.14 ± 0.05++++	0.60 ± 0.08****@@@@	**Diet**<br>**Strain**<br>**Diet X Strain**	48.53<br>52.43<br>0.03	<0.0001<br><0.0001<br>0.86
PC ae C32:2	-2.88 ± 0.08	-1.60 ± 0.09****	-2.40 ± 0.06+++	-1.34 ± 0.09****	**Diet**<br>**Strain**<br>**Diet X Strain**	211.46<br>21.03<br>1.86	<0.0001<br><0.0001<br>0.18
PC ae C34:0	-0.98 ± 0.07	-0.65 ± 0.06*	-0.24 ± 0.09++++	-0.27 ± 0.06@@	**Diet**<br>**Strain**<br>**Diet X Strain**	4.64<br>62.51<br>6.32	0.04<br><0.0001<br>0.02
PC ae C34:1	1.79 ± 0.07	2.94 ± 0.05****	1.88 ± 0.07	2.93 ± 0.10****	**Diet**<br>**Strain**<br>**Diet X Strain**	233.91<br>0.32<br>0.49	<0.0001<br>0.57<br>0.49
PC ae C34:2	1.72 ± 0.08	1.70 ± 0.05	2.26 ± 0.08++++	2.33 ± 0.06@@@@	**Diet**<br>**Strain**<br>**Diet X Strain**	0.09<br>67.52<br>0.44	0.77<br><0.0001<br>0.51
PC ae C34:3					**Diet**	74.40	<0.0001

	-0.10 ± 0.08	0.61 ± 0.07****	0.66 ± 0.06^{++++}	1.27 ± 0.09****$^{@@@@}$	**Strain**<br>**Diet X Strain**	87.13<br>0.45	<0.0001<br>0.51
PC ae C36:0	-1.26 ± 0.08	-0.52 ± 0.06****	-0.83 ± 0.06^{+++}	-0.27 ± 0.07****	**Diet**<br>**Strain**<br>**Diet X Strain**	90.34<br>25.08<br>1.72	<0.0001<br><0.0001<br>0.20
PC ae C36:1	0.64 ± 0.08	2.17 ± 0.05****	1.23 ± 0.07^{++++}	2.41 ± 0.07****	**Diet**<br>**Strain**<br>**Diet X Strain**	256.70<br>28.46<br>1.70	<0.0001<br><0.0001<br>0.20
PC ae C36:2	2.87 ± 0.07	3.26 ± 0.05***	3.80 ± 0.06^{++++}	3.76 ± 0.06$^{@@@@}$	**Diet**<br>**Strain**<br>**Diet X Strain**	8.96<br>144.82<br>12.96	0.004<br><0.0001<br>0.0008
PC ae C36:3	1.02 ± 0.07	1.77 ± 0.05****	1.19 ± 0.07	1.84 ± 0.08****	**Diet**<br>**Strain**<br>**Diet X Strain**	105.32<br>3.23<br>0.52	<0.0001<br>0.08<br>0.47
PC ae C36:4	1.90 ± 0.08	2.34 ± 0.05***	2.20 ± 0.07^{+}	2.53 ± 0.07**	**Diet**<br>**Strain**<br>**Diet X Strain**	31.60<br>12.85<br>0.65	<0.0001<br>0.0008<br>0.42
PC ae C36:5	0.84 ± 0.09	1.32 ± 0.06***	1.34 ± 0.06^{++++}	1.90 ± 0.08****$^{@@@@}$	**Diet**<br>**Strain**<br>**Diet X Strain**	50.30<br>54.23<br>0.32	<0.0001<br><0.0001<br>0.57
PC ae C38:0	1.51 ± 0.10	1.29 ± 0.08	1.30 ± 0.07	1.22 ± 0.07	**Diet**<br>**Strain**<br>**Diet X Strain**	3.29<br>2.84<br>0.74	0.08<br>0.10<br>0.39
PC ae C38:1	-1.85 ± 0.15	-0.69 ± 0.12****	-1.51 ± 0.19	-0.32 ± 0.14****	**Diet**<br>**Strain**<br>**Diet X Strain**	59.06<br>5.36<br>0.003	<0.0001<br>0.03<br>0.96
PC ae C38:2	0.33 ± 0.08	0.07 ± 0.08	1.58 ± 0.07^{++++}	0.71 ± 0.07****$^{@@@@}$	**Diet**<br>**Strain**<br>**Diet X Strain**	56.51<br>156.60<br>16.09	<0.0001<br><0.0001<br>0.0002
PC ae C38:3	-0.20 ± 0.08	0.64 ± 0.06****	0.41 ± 0.05^{++++}	0.92 ± 0.04****$^{@}$	**Diet**<br>**Strain**<br>**Diet X Strain**	120.33<br>51.18<br>7.34	<0.0001<br><0.0001<br>0.01
PC ae C38:4	1.94 ± 0.07	2.38 ± 0.07***	2.64 ± 0.07^{++++}	2.76 ± 0.08$^{@@}$	**Diet**<br>**Strain**	14.93<br>57.20	0.0004<br><0.0001

					**Diet X Strain**	5.05	0.03
PC ae C38:5	1.74 ± 0.07	2.58 ± 0.04****	1.79 ± 0.06	2.65 ± 0.06****	**Diet**	210.03	<0.0001
					**Strain**	1.09	0.30
					**Diet X Strain**	0.06	0.80
PC ae C38:6	0.91 ± 0.08	1.03 ± 0.05	1.59 ± 0.06^{++++}	1.65 ± 0.07$^{@@@@}$	**Diet**	1.84	0.18
					**Strain**	99.78	<0.0001
					**Diet X Strain**	0.16	0.69
PC ae C40:1	1.25 ± 0.06	1.37 ± 0.08	1.22 ± 0.08	1.48 ± 0.05	**Diet**	6.76	0.01
					**Strain**	0.33	0.57
					**Diet X Strain**	0.99	0.33
PC ae C40:2	-1.41 ± 0.06	-1.11 ± 0.06**	-0.81 ± 0.06^{++++}	-0.91 ± 0.04	**Diet**	1.32	0.26
					**Strain**	48.79	<0.0001
					**Diet X Strain**	7.85	0.007
PC ae C40:3	-2.10 ± 0.08	-1.66 ± 0.07***	-1.62 ± 0.08^{++++}	-1.52 ± 0.04	**Diet**	17.06	0.0002
					**Strain**	21.89	<0.0001
					**Diet X Strain**	6.83	0.01
PC ae C40:4	0.40 ± 0.07	0.05 ± 0.08*	1.07 ± 0.08^{++++}	0.42 ± 0.08****$^{@}$	**Diet**	41.17	<0.0001
					**Strain**	43.97	<0.0001
					**Diet X Strain**	3.76	0.06
PC ae C40:5	-0.79 ± 0.09	-0.28 ± 0.06****	-0.30 ± 0.06^{++++}	-0.06 ± 0.05*	**Diet**	36.63	<0.0001
					**Strain**	31.31	<0.0001
					**Diet X Strain**	4.46	0.04
PC ae C40:6	1.06 ± 0.07	0.90 ± 0.05	1.90 ± 0.07^{++++}	1.60 ± 0.09*$^{@@@@}$	**Diet**	10.87	0.002
					**Strain**	119.50	<0.0001
					**Diet X Strain**	0.98	0.33
PC ae C42:0	-2.67 ± 0.09	-2.55 ± 0.12**	-2.22 ± 0.10	-2.40 ± 0.09**	**Diet**	23.53	<0.0001
					**Strain**	3.19	0.08
					**Diet X Strain**	0.003	0.96
PC ae C42:1	-1.30 ± 0.09	-1.15 ± 0.09	-0.96 ± 0.06^{++}	-1.01 ± 0.03	**Diet**	0.45	0.51
					**Strain**	11.85	0.001
					**Diet X Strain**	2.08	0.16
PC ae C42:2	-1.50 ± 0.11	-0.70 ± 0.07****	-1.32 ± 0.08	-0.71 ± 0.05****	**Diet**	75.03	<0.0001
					**Strain**	1.16	0.29
					**Diet X Strain**	1.32	0.26

PC ae C42:3	-0.60 ± 0.10	-0.56 ± 0.07	-0.78 ± 0.05	-0.64 ± 0.10	**Diet**<br>**Strain**<br>**Diet X Strain**	1.19<br>2.46<br>0.31	0.28<br>0.12<br>0.58
PC ae C42:4	-2.74 ± 0.10	-2.82 ± 0.15	-2.46 ± 0.05	-2.77 ± 0.08	**Diet**<br>**Strain**<br>**Diet X Strain**	3.86<br>2.70<br>1.43	0.06<br>0.11<br>0.24
PC ae C42:5	-1.23 ± 0.06	-1.16 ± 0.05	-0.74 ± 0.05++++	-0.97 ± 0.04*	**Diet**<br>**Strain**<br>**Diet X Strain**	0.55<br>37.19<br>3.44	0.46<br><0.0001<br>0.07
PC ae C44:3	-3.07 ± 0.10	-3.02 ± 0.13	-3.20 ± 0.09	-3.04 ± 0.09	**Diet**<br>**Strain**<br>**Diet X Strain**	0.95<br>0.48<br>0.29	0.34<br>0.49<br>0.59
PC ae C44:4	-2.61 ± 0.09	-2.42 ± 0.10	-2.51 ± 0.21	-2.44 ± 0.10	**Diet**<br>**Strain**<br>**Diet X Strain**	1.10<br>0.10<br>0.22	0.30<br>0.75<br>0.64
PC ae C44:5	-2.06 ± 0.08	-1.96 ± 0.12	-2.06 ± 0.08	-2.05 ± 0.07	**Diet**<br>**Strain**<br>**Diet X Strain**	0.37<br>0.33<br>0.27	0.55<br>0.57<br>0.61
PC ae C44:6	-2.41 ± 0.15	-2.84 ± 0.07	-2.22 ± 0.19	-2.44 ± 0.14	**Diet**<br>**Strain**<br>**Diet X Strain**	5.03<br>4.21<br>0.47	0.03<br>0.05<br>0.50
**Sphingolipids**							
SM(OH) C14:1	0.17 ± 0.06	0.87 ± 0.07****	0.95 ± 0.06++++	1.72 ± 0.04****@@@@	**Diet**<br>**Strain**<br>**Diet X Strain**	153.01<br>190.26<br>0.34	<0.0001<br><0.0001<br>0.56
SM(OH) C16:1	-0.89 ± 0.06	-0.50 ± 0.05****	-0.38 ± 0.06++++	0.14 ± 0.05****@@@@	**Diet**<br>**Strain**<br>**Diet X Strain**	73.57<br>116.19<br>1.55	<0.0001<br><0.0001<br>0.22
SM(OH) C22:1	1.15 ± 0.08	1.56 ± 0.06***	2.04 ± 0.05++++	2.18 ± 0.07@@@@	**Diet**<br>**Strain**<br>**Diet X Strain**	16.63<br>124.66<br>3.83	0.0002<br><0.0001<br>0.06
SM(OH) C22:2					**Diet**	57.77	<0.0001

	2.28 ± 0.07	1.96 ± 0.07*	3.41 ± 0.08^{++++}	2.69 ± 0.05****$^{@@@@}$	**Strain**<br>**Diet X Strain**	184.09<br>8.81	<0.0001<br>0.005
SM(OH) C24:1	0.53 ± 0.07	0.64 ± 0.07	0.42 ± 0.07	0.65 ± 0.08	**Diet**<br>**Strain**<br>**Diet X Strain**	5.72<br>0.50<br>0.77	0.02<br>0.48<br>0.38
SM C16:0	4.10 ± 0.06	5.03 ± 0.06****	4.58 ± 0.04^{++++}	5.43 ± 0.04****$^{@@@@}$	**Diet**<br>**Strain**<br>**Diet X Strain**	306.49<br>76.63<br>0.66	<0.0001<br><0.0001<br>0.42
SM C16:1	1.88 ± 0.06	2.35 ± 0.04****	2.53 ± 0.05^{++++}	2.96 ± 0.07****$^{@@@}$	**Diet**<br>**Strain**<br>**Diet X Strain**	64.96<br>124.59<br>0.13	<0.0001<br><0.0001<br>0.72
SM C18:0	2.85 ± 0.09	3.62 ± 0.06****	2.95 ± 0.12	3.43 ± 0.10**	**Diet**<br>**Strain**<br>**Diet X Strain**	43.44<br>0.19<br>2.21	<0.0001<br>0.66<br>0.14
SM C18:1	0.64 ± 0.04	0.98 ± 0.06**	0.69 ± 0.07	1.21 ± 0.07****	**Diet**<br>**Strain**<br>**Diet X Strain**	48.73<br>5.22<br>2.14	<0.0001<br>0.03<br>0.15
SM C20:2	-0.33 ± 0.07	-0.38 ± 0.08	-0.78 ± 0.09^{++}	-1.09 ± 0.10$^{@@@@}$	**Diet**<br>**Strain**<br>**Diet X Strain**	4.40<br>45.10<br>2.26	0.04<br><0.0001<br>0.14
SM C22:3	-2.63 ± 0.07	-2.17 ± 0.08***	-2.37 ± 0.06	-2.18 ± 0.09	**Diet**<br>**Strain**<br>**Diet X Strain**	18.72<br>2.87<br>3.46	<0.0001<br>0.10<br>0.07
SM C24:0	3.05 ± 0.11	3.69 ± 0.06****	3.81 ± 0.08^{++++}	3.90 ± 0.07	**Diet**<br>**Strain**<br>**Diet X Strain**	20.53<br>35.14<br>11.47	<0.0001<br><0.0001<br>0.001
SM C24:1	4.29 ± 0.08	4.18 ± 0.14	488 ± 0.05^{+++}	4.63 ± 0.07$^{@@}$	**Diet**<br>**Strain**<br>**Diet X Strain**	4.22<br>34.12<br>0.72	0.05<br><0.0001<br>0.40
SM C26:0	-2.07 ± 0.09	-1.94 ± 0.08	-1.80 ± 0.09	-1.87 ± 0.10	**Diet**<br>**Strain**<br>**Diet X Strain**	0.11<br>3.29<br>1.28	0.75<br>0.08<br>0.26
SM C26:1	-2.42 ± 0.13	-2.32 ± 0.10	-1.92 ± 0.14^{+}	-2.21 ± 0.13	**Diet**<br>**Strain**	0.58<br>6.05	0.45<br>0.02

					**Diet X Strain**	2.53	0.12
**Monosaccharides**							
Hexoses (H1)	12.79 ± 0.23	13.28 ± 0.07	13.05 ± 0.17	12.78 ± 0.14	**Diet**<br>**Strain**<br>**Diet X Strain**	0.46<br>0.48<br>5.08	0.50<br>0.49<br>0.03
**Metabolite ratios**							
Short chain acylcarnitines (SCACs)	3.85 ± 0.07	2.89 ± 0.08****	4.53 ± 0.16+++	2.96 ± 0.12****	**Diet**<br>**Strain**<br>**Diet X Strain**	122.43<br>10.78<br>7.12	<0.0001<br>0.002<br>0.01
Medium chain acylcarnitines (MCACs)	-0.33 ± 0.05	-0.31 ± 0.05	-0.25 ± 0.03	-0.31 ± 0.05	**Diet**<br>**Strain**<br>**Diet X Strain**	0.16<br>0.94<br>0.95	0.69<br>0.34<br>0.34
Ling chain acylcarnitines (LCACs)	0.34 ± 0.05	0.22 ± 0.04	0.72 ± 0.14+	0.35 ± 0.07*	**Diet**<br>**Strain**<br>**Diet X Strain**	8.00<br>8.78<br>2.01	0.007<br>0.005<br>0.16
(C2+C3) / C0	-0.94 ± 0.09	-1.02 ± 0.06	-0.25 ± 0.25+	-0.63 ± 0.18	**Diet**<br>**Strain**<br>**Diet X Strain**	1.86<br>10.15<br>0.77	0.18<br>0.003<br>0.38
C3/C4	0.52 ± 0.08	0.79 ± 0.05	0.39 ± 0.08	0.91 ± 0.08****	**Diet**<br>**Strain**<br>**Diet X Strain**	30.92<br>0.01<br>3.25	<0.0001<br>0.94<br>0.08
C4/C0	-5.55 ± 0.11***	-4.87 ± 0.07	-5.15 ± 0.15	-4.50 ± 0.09***	**Diet**<br>**Strain**<br>**Diet X Strain**	36.50<br>11.93<br>0.02	<0.0001<br>0.001<br>0.89
C4/C5	0.28 ± 0.09**	-0.19 ± 0.07	0.53 ± 0.09	-0.23 ± 0.09****	**Diet**<br>**Strain**<br>**Diet X Strain**	49.53<br>1.47<br>2.62	<0.0001<br>0.23<br>0.11
C5/C0	-5.83 ± 0.08****	-4.68 ± 0.12	-5.68 ± 0.13	-4.27 ± 0.12****	**Diet**<br>**Strain**<br>**Diet X Strain**	126.77<br>5.99<br>1.20	<0.0001<br>0.02<br>0.28

C3/C0	-5.03 ± 0.07****	-4.08 ± 0.10	-4.77 ± 0.11++	-3.59 ± 0.11****	**Diet**<br>**Strain**<br>**Diet X Strain**	120.14<br>15.19<br>1.32	0.00<br>0.0003<br>0.26
C18/C18:1	-0.52 ± 0.14*	0.003 ± 0.10	-0.50 ± 0.10	0.01 ± 0.09**	**Diet**<br>**Strain**<br>**Diet X Strain**	22.24<br>0.01<br>0.001	<0.0001<br>0.93<br>0.98
(C16 + C18)/C0<br>(CPT-1)	-6.73 ± 0.08	-5.55 ± 0.10***	-6.05 ± 0.30+	-4.75 ± 0.15****	**Diet**<br>**Strain**<br>**Diet X Strain**	44.50<br>15.97<br>0.09	<0.0001<br>0.0002<br>0.77
(C16 + C18:1)/C2<br>(CPT-2)	-5.55 ± 0.10	-4.34 ± 0.10****	-5.61 ± 0.11	-3.90 ± 0.14****	**Diet**<br>**Strain**<br>**Diet X Strain**	164.80<br>2.75<br>4.80	<0.0001<br>0.10<br>0.03
C2/C0	-1.03 ± 0.09	-1.21 ± 0.07	-0.32 ± 0.26+	-0.86 ± 0.21	**Diet**<br>**Strain**<br>**Diet X Strain**	3.99<br>8.62<br>0.95	0.05<br>0.005<br>0.34
Total AC / C0	-0.46 ± 0.07	-0.11 ± 0.05	0.12 ± 0.23+	0.29 ± 0.13	**Diet**<br>**Strain**<br>**Diet X Strain**	3.32<br>11.44<br>0.38	0.07<br>0.001<br>0.54
Total AC-DC / Total AC	-4.03 ± 0.07	-3.21 ± 0.07****	-4.54 ± 0.15++	-3.27 ± 0.11****	**Diet**<br>**Strain**<br>**Diet X Strain**	99.11<br>7.45<br>4.65	<0.0001<br>0.009<br>0.04
Total AC-OH / Total AC	-4.94 ± 0.06	-4.20 ± 0.07****	-5.46 ± 0.12+++	-4.35 ± 0.10****	**Diet**<br>**Strain**<br>**Diet X Strain**	100.32<br>13.90<br>4.39	<0.0001<br>0.0005<br>0.04
Total AC	4.04 ± 0.07	3.24 ± 0.06****	4.68 ± 0.15+++	3.31 ± 0.10****	**Diet**<br>**Strain**<br>**Diet X Strain**	108.64<br>11.60<br>7.23	<0.0001<br>0.001<br>0.01
Total AC-DC	0.02 ± 0.04	0.03 ± 0.03	0.14 ± 0.05	0.04 ± 0.04	**Diet**<br>**Strain**<br>**Diet X Strain**	0.99<br>2.67<br>1.65	0.32<br>0.11<br>0.21
Total AC-OH	-0.89 ± 0.04	-0.97 ± 0.03	-0.79 ± 0.04	-1.04 ± 0.04***	**Diet**<br>**Strain**<br>**Diet X Strain**	17.20<br>0.08<br>4.90	0.0001<br>0.78<br>0.03
					**Diet**	14.50	0.0004

Branched-chain amino acids (BCAA)	8.56 ± 0.08	9.00 ± 0.19	9.23 ± 0.08^{++}	9.76 ± 0.15$^{*@@@}$	**Strain**<br>**Diet X Strain**	30.83<br>0.14	<0.0001<br>0.71
Aromatic amino acids (AAA)	7.59 ± 0.08	7.47 ± 0.10	7.83 ± 0.07	7.83 ± 0.11	**Diet**<br>**Strain**<br>**Diet X Strain**	0.46<br>10.57<br>0.52	0.50<br>0.002<br>0.47
BCAA/AAA	0.96 ± 0.05	1.53 ± 0.10***	1.40 ± 0.07^{++}	1.94 ± 0.13***$^{@}$	**Diet**<br>**Strain**<br>**Diet X Strain**	36.18<br>20.82<br>0.04	<0.0001<br><0.0001<br>0.84
Gly/Glu	0.42 ± 0.22	0.31 ± 0.17	0.58 ± 0.14	-0.30 ± 0.19**	**Diet**<br>**Strain**<br>**Diet X Strain**	7.61<br>1.56<br>4.78	0.008<br>0.22<br>0.03
Glu/His	1.52 ± 0.24	1.06 ± 0.18	1.41 ± 0.14	1.41 ± 0.20	**Diet**<br>**Strain**<br>**Diet X Strain**	1.44<br>0.41<br>1.39	0.24<br>0.53<br>0.24
Gly/Ser	1.15 ± 0.08	0.83 ± 0.11	0.84 ± 0.09	0.39 ± 0.10**$^{@}$	**Diet**<br>**Strain**<br>**Diet X Strain**	17.25<br>16.29<br>0.48	0.0001<br>0.0002<br>0.49
Tyr/Phe	-0.12 ± 0.09	0.10 ± 0.07	-0.37 ± 0.08	0.005 ± 0.11*	**Diet**<br>**Strain**<br>**Diet X Strain**	12.26<br>4.14<br>0.77	0.001<br>0.05<br>0.38
Arg/Cit	0.79 ± 0.11	0.42 ± 0.13	1.09 ± 0.17	0.61 ± 0.12	**Diet**<br>**Strain**<br>**Diet X Strain**	9.96<br>3.21<br>0.15	0.003<br>0.08<br>0.70
Glucogenic amino acids	11.02 ± 0.06	10.96 ± 0.10	11.07 ± 0.06	11.10 ± 0.09	**Diet**<br>**Strain**<br>**Diet X Strain**	0.02<br>1.42<br>0.28	0.89<br>0.24<br>0.60
Ketogenic amino acids	8.28 ± 0.09	8.72 ± 0.15	9.08 ± 0.11^{+++}	9.11 ± 0.12	**Diet**<br>**Strain**<br>**Diet X Strain**	3.85<br>24.95<br>3.03	0.06<br><0.0001<br>0.09
Glucogenic + Ketogenic amino acids	8.07 ± 0.08	8.11 ± 0.13	8.47 ± 0.06^{+}	8.68 ± 0.11$^{@@}$	**Diet**<br>**Strain**<br>**Diet X Strain**	1.68<br>23.81<br>0.68	0.20<br><0.0001<br>0.41
Essential amino acids	9.87 ± 0.07	10.10 ± 0.14	10.30 ± 0.08^{+}	10.57 ± 0.11$^{@}$	**Diet**<br>**Strain**	6.14<br>19.16	0.02<br><0.0001

					**Diet X Strain**	0.04	0.84
Non-essential amino acids	10.74 ± 0.07	10.63 ± 0.10	10.81 ± 0.06	10.67 ± 0.10	**Diet**	2.02	0.16
					**Strain**	0.39	0.54
					**Diet X Strain**	0.03	0.86
Total DMA	-3.47 ± 0.44	-4.17 ± 0.45	-2.72 ± 0.33	-3.66 ± 0.30	**Diet**	4.55	0.04
					**Strain**	2.70	0.11
					**Diet X Strain**	0.10	0.76
Spermidine/putrescine	1.35 ± 0.14	1.05 ± 0.10	2.09 ± 0.16++	1.95 ± 0.13@@@	**Diet**	2.64	0.11
					**Strain**	37.19	<0.0001
					**Diet X Strain**	0.35	0.56
Kynurenin/tryptophan	-5.90 ± 0.11	-6.48 ± 0.12**	-5.74 ± 0.10	-6.22 ± 0.12*	**Diet**	22.69	<0.0001
					**Strain**	3.52	0.07
					**Diet X Strain**	0.21	0.65
Serotonin/tryptophan	-3.01 ± 0.46	-2.28 ± 0.31	-3.32 ± 0.19	-2.99 ± 0.21	**Diet**	3.44	0.08
					**Strain**	3.19	0.09
					**Diet X Strain**	0.51	0.48
Serotonin/kynurenine	2.75 ± 0.04	4.22 ± 0.31	2.47 ± 0.25	3.18 ± 0.24	**Diet**	12.49	0.002
					**Strain**	4.61	0.04
					**Diet X Strain**	1.49	0.24
Met/Met-SO	5.76 ± 0.48	4.97 ± 0.62	5.01 ± 0.14	4.31 ± 0.24	**Diet**	3.80	0.06
					**Strain**	3.33	0.08
					**Diet X Strain**	0.01	0.91
ADMA/Arg	-7.64 ± 0.14	-7.72 ± 0.20	-7.94 ± 0.17	-7.49 ± 0.24	**Diet**	0.92	0.34
					**Strain**	0.03	0.87
					**Diet X Strain**	1.85	0.18
Kynurenine/Alpha-amioadipate	-1.45 ± 0.17	-2.63 ± 0.17****	-0.30 ± 0.09+++	-0.97 ± 0.19@@@@	**Diet**	25.57	<0.0001
					**Strain**	59.61	<0.0001
					**Diet X Strain**	2.00	0.17
Leucine/kynurenine	6.77 ± 0.11	7.99 ± 0.13****	7.00 ± 0.11	8.13 ± 0.12****	**Diet**	102.68	<0.0001
					**Strain**	2.57	0.12
					**Diet X Strain**	0.14	0.71
SFA aa	4.28 ± 0.09	4.55 ± 0.06	4.74 ± 0.12++	4.54 ± 0.10	**Diet**	0.13	0.72
					**Strain**	5.68	0.02
					**Diet X Strain**	6.22	0.02

PUFA aa	9.95 ± 0.07	10.72 ± 0.06****	10.28 ± 0.05^{++}	10.80 ± 0.06****	**Diet**<br>**Strain**<br>**Diet X Strain**	116.00<br>12.32<br>4.15	<0.0001<br>0.001<br>0.05
MUFA aa	7.22 ± 0.10	9.25 ± 0.08****	7.33 ± 0.10	9.15 ± 0.14****	**Diet**<br>**Strain**<br>**Diet X Strain**	320.5<br>0.004<br>1.07	<0.0001<br>0.95<br>0.31
UFA aa	10.16 ± 0.07	11.17 ± 0.06****	10.46 ± 0.05^{++}	11.21 ± 0.08****	**Diet**<br>**Strain**<br>**Diet X Strain**	193.4<br>7.76<br>4.17	<0.0001<br>0.008<br>0.05
Total PC aa	10.18 ± 0.07	11.18 ± 0.06****	10.49 ± 0.05^{++}	11.23 ± 0.08****	**Diet**<br>**Strain**<br>**Diet X Strain**	191.90<br>8.02<br>4.35	<0.0001<br>0.007<br>0.04
SFA ae	2.20 ± 0.07	2.16 ± 0.06	2.25 ± 0.05	2.25 ± 0.05	**Diet**<br>**Strain**<br>**Diet X Strain**	0.09<br>1.35<br>0.08	0.76<br>0.25<br>0.78
PUFA ae	5.24 ± 0.06	5.62 ± 0.04****	5.84 ± 0.06^{++++}	5.99 ± 0.05$^{@@@@}$	**Diet**<br>**Strain**<br>**Diet X Strain**	24.46<br>83.45<br>4.73	<0.0001<br><0.0001<br>0.04
MUFA ae	3.16 ± 0.06	4.09 ± 0.05****	3.39 ± 0.05^{+}	4.18 ± 0.05****	**Diet**<br>**Strain**<br>**Diet X Strain**	283.2<br>9.59<br>1.85	<0.0001<br>0.003<br>0.18
UFA ae	5.55 ± 0.06	6.05 ± 0.04****	6.09 ± 0.05^{++++}	6.38 ± 0.06**$^{@@@}$	**Diet**<br>**Strain**<br>**Diet X Strain**	56.40<br>67.18<br>4.08	<0.0001<br><0.0001<br>0.05
Total PC ae	5.68 ± 0.06	6.15 ± 0.04****	6.19 ± 0.05^{++++}	6.46 ± 0.06**$^{@@@}$	**Diet**<br>**Strain**<br>**Diet X Strain**	49.67<br>61.01<br>3.30	<0.0001<br><0.0001<br>0.07
SFA (PC ae + PC aa)	4.59 ± 0.07	4.81 ± 0.06	4.99 ± 0.10^{++}	4.81 ± 0.09	**Diet**<br>**Strain**<br>**Diet X Strain**	0.06<br>5.79<br>5.42	0.81<br>0.02<br>0.02
PUFA (PC ae + PC aa)	10.01 ± 0.07	10.76 ± 0.06****	10.35 ± 0.05^{+++}	10.85 ± 0.06****	**Diet**<br>**Strain**<br>**Diet X Strain**	112.6<br>13.70<br>4.28	<0.0001<br>0.0006<br>0.04
MUFA (PC ae + PC aa)					**Diet**	319.40	<0.0001

	7.30 ± 0.10	9.29 ± 0.08****	7.43 ± 0.09	9.19 ± 0.14****	**Strain**<br>**Diet X Strain**	0.02<br>1.10	0.89<br>0.30
UFA (PC ae + PC aa)	10.21 ± 0.07	11.21 ± 0.06****	10.53 ± 0.05^{++}	11.26 ± 0.08****	**Diet**<br>**Strain**<br>**Diet X Strain**	188.90<br>8.74<br>4.29	<0.0001<br>0.005<br>0.04
Total LysoPC	8.46 ± 0.07	8.97 ± 0.08****	8.42 ± 0.05	8.86 ± 0.09***	**Diet**<br>**Strain**<br>**Diet X Strain**	42.68<br>1.21<br>0.24	<0.0001<br>0.28<br>0.63
lysoPC a C16:1/<br>lysoPC a C16:0	-4.62 ± 0.11	-4.81 ± 0.09	-5.63 ± 0.07^{++++}	-5.59 ± 0.07$^{@@@@}$	**Diet**<br>**Strain**<br>**Diet X Strain**	0.79<br>104.1<br>1.65	0.38<br><0.0001<br>0.21
lysoPC a C18:2/<br>lysoPC a C18:1	1.29 ± 0.06	0.43 ± 0.06****	1.69 ± 0.05^{++}	0.70 ± 0.13****	**Diet**<br>**Strain**<br>**Diet X Strain**	132.9<br>17.86<br>0.72	<0.0001<br>0.0001<br>0.40
lysoPC a C20:4/<br>lysoPC a C20:3	2.06 ± 0.10	0.55 ± 0.08****	2.55 ± 0.08^{++}	1.09 ± 0.12****$^{@@}$	**Diet**<br>**Strain**<br>**Diet X Strain**	243.6<br>29.88<br>0.09	<0.0001<br><0.0001<br>0.76
Total SM	6.12 ± 0.06	6.59 ± 0.06****	6.69 ± 0.05^{++++}	6.94 ± 0.04*$^{@@@}$	**Diet**<br>**Strain**<br>**Diet X Strain**	46.73<br>76.91<br>4.68	<0.0001<br><0.0001<br>0.04
Total SM-non-OH	5.89 ± 0.06	6.41 ± 0.06****	6.40 ± 0.05^{++++}	6.72 ± 0.04***$^{@@}$	**Diet**<br>**Strain**<br>**Diet X Strain**	63.55<br>59.02<br>3.76	<0.0001<br><0.0001<br>0.06
Total SM-OH	3.35 ± 0.06	3.45 ± 0.05	4.23 ± 0.06^{++++}	4.08 ± 0.05$^{@@@@}$	**Diet**<br>**Strain**<br>**Diet X Strain**	0.18<br>179.10<br>4.93	0.67<br><0.0001<br>0.03
Total SM-OH /<br>Total SM-non-OH	-2.53 ± 0.03	-2.96 ± 0.03****	-2.16 ± 0.04^{++++}	-2.64 ± 0.03****$^{@@@@}$	**Diet**<br>**Strain**<br>**Diet X Strain**	172.0<br>102.6<br>0.42	<0.0001<br><0.0001<br>0.52

Supplementary Table S2. Significantly different metabolites in Bl6 and 129Sv with standard and HFD. The Bonferroni correction was applied (p<0.00027) to compare two mouse strains.

Standard food			High fat diet		
Metabolite	*t*-value	adjusted *p*-value	Metabolite	*t*-value	adjusted *p*-value
**Higher in Bl6**					
LysoPC a C16:1	6.20	0.000003	LysoPC a C16:1	5.34	0.00002
LysoPC a C20:3	4.71	0.0001	LysoPC a C20:3	4.45	0.0002
Carnosine	8.26	<0.000001	Alpha-AAA	6.24	0.00002
			Carnosine	5.98	0.000005
			Putrescine	5.59	0.000005
			t4-OH-Pro	8.38	<0.000001
			PC aa C32:1	4.68	0.0001
			PC aa C34:3	5.26	0.00003
			SM C20:2	5.48	0.00002

Higher in 129Sv					
C3	-4.34	0.0002	PC aa C30:2	-4.90	0.00007
LysoPC a C17:0	-6.77	0.000001	PC aa C40:5	-4.60	0.0001
LysoPC a C24:0	-5.27	0.00002	PC ae C32:1	-4.44	0.0002
Ile	-6.19	0.000003	PC ae C34:0	-4.43	0.0002
Leu	-5.66	0.000009	PC ae C34:2	-7.71	<0.000001
Lys	-5.19	0.00003	PC ae C34:3	-5.85	0.000007
Orn	-4.52	0.0002	PC ae C36:2	-6.32	0.000002
Ser	-4.83	0.00007	PC ae C36:5	-5.96	0.000005
Val	-5.72	0.000008	PC ae C38:2	-6.16	0.000003
PC aa C30:2	-5.22	0.00003	PC ae C38:6	-7.29	<0.000001
PC aa C36:0	-5.15	0.00003	PC ae C40:6	-6.84	0.000001
PC aa C36:2	-6.47	0.000001	SM (OH) C14:1	-10.33	<0.000001
PC aa C40:2	-4.88	0.00006	SM (OH) C16:1	-9.73	<0.000001
PC aa C40:5	-4.36	0.0002	SM (OH) C22:1	-6.49	0.000002
PC aa C40:6	-5.05	0.00004	SM (OH) C22:2	-8.34	<0.000001
PC ae C30:2	-7.32	<0.000001	SM C16:0	-5.47	0.00002
PC ae C32:1	-6.05	0.000004	SM C16:1	-7.57	<0.000001

PC ae C32:2	-4.67	0.0001	CPT-1	-4.52	0.0002
PC ae C34:0	-6.63	0.000001	Spermidine/Putrescine	-5.53	0.00002
PC ae C34:2	-4.67	0.0001	Kynurenine/Alpha-AAA	-5.76	0.00004
PC ae C34:3	-7.42	<0.000001			
PC ae C36:1	-5.63	0.00001			
PC ae C36:2	-10.54	<0.000001			
PC ae C36:5	4.57	0.0001			
PC ae C38:2	-11.44	<0.000001			
PC ae C38:3	-6.42	0.000001			
PC ae C38:4	-7.38	<0.000001			
PC ae C38:6	6.94	<0.000001			
PC ae C40:2	7.05	<0.000001			
PC ae C40:3	-4.53	0.0001			
PC ae C40:4	-6.26	0.000002			
PC ae C40:5	-4.76	0.00008			

PC ae C40:6	-8.66	<0.000001			
PC ae C42:5	-6.10	0.000003			
SM (OH) C14:1	-9.22	<0.000001			
SM (OH) C16:1	-6.11	0.000003			
SM (OH) C22:1	-9.31	<0.000001			
SM (OH) C22:2	-10.79	<0.000001			
SM C16:0	-6.94	<0.000001			
SM C16:1	-8.22	<0.000001			
SM C24:0	-5.74	0.000008			
SM C24:1	-6.53	0.000001			
BCAA	-6.14	0.000003			
BCAA/AAA	5.14	0.00003			
Ketogenic amino acids	-5.61	0.00001			
Kynurenine/ Alpha-AAA	-5.04	0.00009			

Supplementary Figure S1. Locomotor activity of Bl6 and 129Sv mice in the first three hours of 24 h Phenotyper trial (Log2 values, data expressed as mean ± SEM). Total distance traveled in the 1st hour of day 1 (A) and week 9 (G), 2nd hour of day 1 (B) and week 9 (H) and 3rd hour of day 1 (C) and week 9 (I). Time spent in the center zone in the 1st hour of day 1 (D) and week 9 (J), 2nd hour of day 1 (E) and week 9 (F) and 3rd hour of day 1 (K) and week 9 (L). Two-way ANOVA (Bonferroni post hoc test): **$p \leq 0.01$ (diet effect), +$p \leq 0.05$, ++$p \leq 0.01$, +++$p \leq 0.001$, ++++$p \leq 0.0001$ (strain effect).

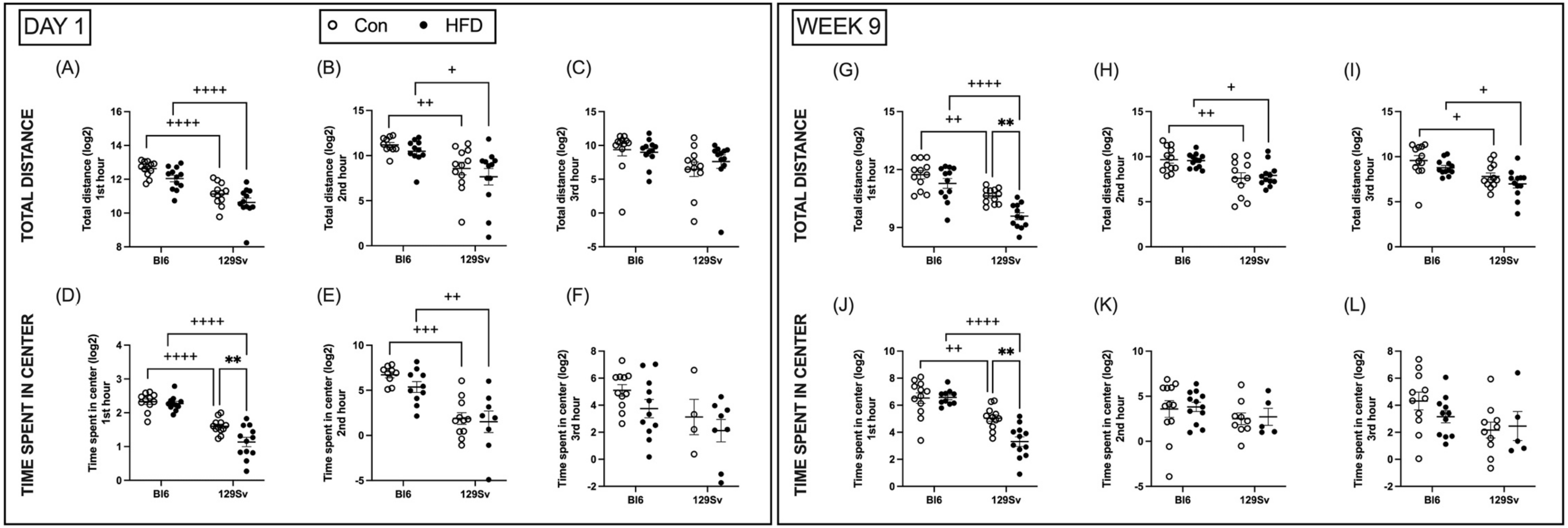

Supplement: Supplementary file 1 [file ijms-23-11682-s001.zip › ijms-1913825-supplementary.pdf]
